# Detailed analysis of hydrocephalus patterns and associated variables in patients after open fetal repair and postnatal myelomeningocele/myeloschisis closure

**DOI:** 10.1007/s00381-025-06819-z

**Published:** 2025-04-16

**Authors:** Adam J. Kundishora, Kamila Bond, Martin Rosenfeld, Sierra D. Land, Taryn Gallagher, Tom A. Reynolds, Juliana S. Gebb, N. Scott Adzick, Tracy M. Flanders, Gregory G. Heuer

**Affiliations:** 1 Division of Neurosurgery, Department of Surgery, Children’s Hospital of Philadelphia, 3501 Civic Center Boulevard, 10 th Floor, Hub for Clinical Collaboration, Philadelphia, PA USA; 2 Department of Neurosurgery, University of Pennsylvania Perlman School of Medicine, Philadelphia, PA USA; 3 Richard D. Wood Jr. Center for Fetal Diagnosis and Treatment, Children’s Hospital of Philadelphia, Philadelphia, PA USA; 4 Division of Pediatric General, Thoracic, and Fetal Surgery, Children’s Hospital of Philadelphia, Philadelphia, PA USA; 5 Department of Surgery, University of Pennsylvania Perlman School of Medicine, Philadelphia, PA USA

**Keywords:** Fetal repair, Myelomeningocele, Myeloschisis, Hydrocephalus, Spina bifida

## Abstract

**Background:**

Myelomeningocele (MMC) and myeloschisis (MS) are severe neural tube defects that result in neurodevelopmental impairments and hydrocephalus due to prenatal spinal cord exposure to amniotic fluid. Fetal MMC/MS (fMMC/MS) repair has become the standard of care in appropriately selected patients, demonstrating improved outcomes, including a reduction in the need for cerebrospinal fluid (CSF) diversion compared to postnatal MMC/MS (pMMC/MS) closure. This study is a detailed analysis of the incidence, timing, and imaging predictors of hydrocephalus and CSF diversion dependence in fetal repair and postnatal closure patients in the post-MOMS trial era.

**Methods:**

A retrospective review was conducted of MMC/MS patients treated at a single institution between 2016 and 2023. Inclusion criteria required complete prenatal and postnatal follow-up data. Imaging metrics, including prenatal atrial diameter (AD) and postnatal fronto-occipital horn ratio (FOR), were analyzed alongside head circumference (HC) growth trajectories. Statistical analyses, including Youden’s index, were performed to identify predictive cutoffs for shunt dependence.

**Results:**

Among 333 MMC/MS patients, fetal repair significantly reduced permanent CSF diversion rates compared to postnatal closure (27.8% vs. 70.1%, *p* < 0.01). Timing of clinical hydrocephalus onset was delayed in fetal patients (24.2 weeks vs. 2.8 weeks, *p* < 0.01). HC of fetal patients was highly correlated with timing of shunt dependence. AD ≥ 10 mm and postnatal FOR ≥ 0.5 were associated with higher shunt dependence (*p* < 0.01). Optimal cut points for predicting shunt dependence were identified by Youden’s index as 14 mm for AD and 0.57 for early postnatal FOR.

**Conclusion:**

Fetal repair of MMC/MS decreases the incidence of clinical hydrocephalus and delays its onset compared to postnatal closure. Early imaging metrics (AD and FOR) may stratify hydrocephalus risk, enabling improved prenatal counseling and postnatal care. Long-term follow-up remains crucial for early detection and management of hydrocephalus in fetal MMC/MS patients.

## Introduction

Open spina bifida due to myelomeningocele (MMC)/myeloschisis (MS) occurs due to failed dorsal fusion of the neural tube during embryonic development [1]. This leads to spinal cord and meningeal protrusion through an opening in the bony elements of the dorsal spinal column. When the delicate spinal cord is exposed in utero, it undergoes cellular degeneration induced by both mechanical trauma and caustic amniotic fluid [2–4]. As such, patients born with open spina bifida face numerous neurological challenges including weakness, numbness, bowel and bladder dysfunction, and cognitive impairments.

In the USA, MMC/MS occurs in approximately three in every 10,000 live births [5]. The morbidity associated with MMC/MS requires the support of complex medical, surgical, and rehabilitative services. Coordinating the resources to provide comprehensive care to this population throughout their lifespan can place a considerable burden on patients, families, and healthcare systems alike [6, 7].

Historically, MMC could only be treated by postnatal surgical closure within the first few days of life. However, this approach was unable to reverse the cellular neuronal damage that had already accumulated over the course of gestation. The advent of fetal MMC (fMMC)/MS (fMS) repair resulted in a change to the care of many patients with open spina bifida. This method, studied in a randomized, controlled fashion in the Management of Myelomeningocele Study (MOMS), demonstrated lower rates of shunt placement, improved functionality, and a lower incidence of hindbrain herniation [8–12].

Since the end of the MOMS trial, the care of MMC/MS patients has continued to evolve. Therefore, ongoing analysis is required to predict patient outcomes and properly counsel families. With prior studies demonstrating lower quality of life scores for open spina bifida patients requiring CSF diversion, understanding associated hydrocephalus is particularly important [13, 14]. This study is a detailed analysis of the incidence and timing of hydrocephalus development for patients who underwent fetal repair versus postnatal closure, specifically as they relate to routine prenatal and postnatal imaging and other clinical variables for MMC/MS.

## Objective

This study aimed to report one institution’s experience on the incidence, timing, and radiographic predictability of hydrocephalus development and shunt dependence after fetal MMC/MS repair in the post-MOMS trial era.

## Methods

The study was approved by our local Institutional Review Board, and all data were stored within Clinical Outcomes Data Archive (CODA) (IRB 21–018553). This is a retrospective review of all patients who underwent fetal repair or postnatal closure of MMC/MS at a single institution between January 2016 and January 2023. Patients with intrauterine fetal demise (IUFD), no postnatal follow-up, or genetic anomalies impacting cranial development were excluded.

Data were abstracted from the electronic medical record using the CODA system [15]. Bony defect level and atrial diameter (AD) of the ventricles were determined by a fetal radiologist using prenatal ultrasound and fetal ultrafast magnetic resonance imaging (MRI), respectively. Fronto-occipital horn ratio (FOR) was calculated from a postnatal cranial ultrasound within the first 7 days of life. Head circumference (HC) *z*-score and percentiles were calculated using World Health Organization (WHO) Child Growth Standards, adjusted for age and sex. The primary outcome variable, cerebrospinal fluid (CSF) diversion, was defined as placement of ventriculoperitoneal/atrial shunt (VP shunt) or endoscopic third ventriculostomy (ETV). Decision between ETV or shunt placement was made considering the ETV success score, anatomic considerations on MRI, and parent preference. In cases of ETV, choroid plexus cauterization was not performed as this is not the practice in the MMC population at our institution.

Continuous variables were summarized as mean (standard deviation; SD) for parametric data and median (interquartile range; IQR) for nonparametric data. Categorical data were summarized as frequencies. Chi-squared and Fisher’s exact tests were used to evaluate associations between CSF diversion and categorical variables. Student’s two-sided *t*-test or ANOVA was used to evaluate associations with normally distributed continuous variables. The Mann–Whitney *U* or Kruskal–Wallis tests were used for covariates that were assessed to not follow a normal distribution. Bonferroni correction was used in pairwise analyses between FOR groups to adjust for multiple comparisons. Receiver operating characteristic (ROC) curves with area under the curve (AUC) were created, and Youden’s index (*J* statistic, defined as sensitivity + specificity − 1) was calculated to select the optimal cutoff point of AD and FOR as a predictor of the need for CSF diversion. Statistical analysis was performed using RStudio 2024.04.01 version 4.3.2 (RStudio Team (2020). RStudio: Integrated Development for R. RStudio, PBC, Boston, MA URL http://www.rstudio.com/).

## Results

### Patient characteristics

**Fig. 1:**
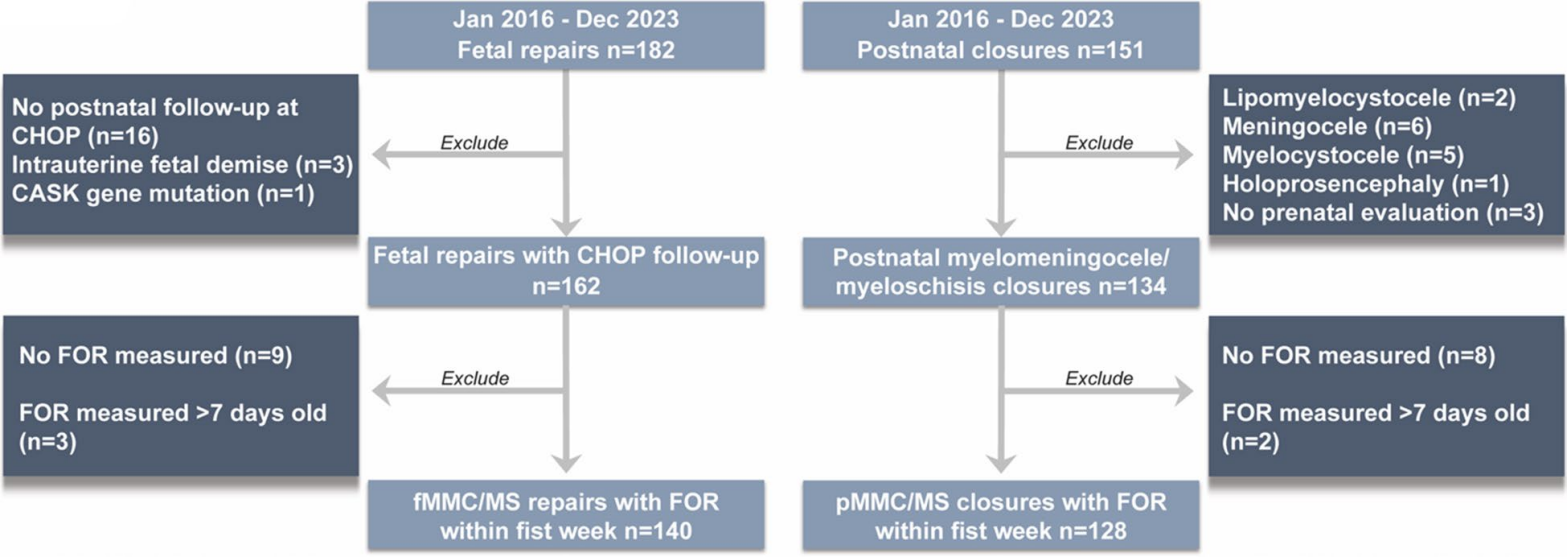
Fetal repair and postnatal closure cohort generation

Of the 333 patients who underwent evaluation for MMC/MS between January 2016 and January 2023 (56% male), 182 were fetal repairs (52% male), and 151 were postnatal (62% male; Table 1). Of the 182 fetal repairs, 166 (91%) had postnatal follow-up. Three cases resulted in IUFD, and one patient was excluded due to a *CASK* gene mutation, bringing the fMMC/MS cohort size to 162 (Fig. 1). Nineteen patients had no FOR measurements recorded at CHOP, and three had FOR measurements at greater than 7 days old. One patient was unable to complete a fetal MRI due to the presence of metal in the mother’s spine (Fig. 1).

**Table 1:** Characteristics of myelomeningocele/myeloschisis closure patients

	Fetal repair			Postnatal closure			Total	
	Female (*n* = 77)	Male (*n* = 85)	Overall (*n* = 162)	Female (*n* = 52)	Male (*n* = 82)	Overall (*n* = 134)	*n* = 296	*p* value
Ventriculo/craniometrics (mean avg.)	mm (SD)			mm (SD)				
Prenatal AD on MRI	12.2 (3.43)	11.4 (3.26)	11.8 (3.36)	12.3 (5.67)	12.4 (4.76)	12.4 (5.10)	12.3 (4.11)	0.27
Prenatal AD on US	11.0 (3.42)	10.2 (3.03)	10.6 (3.24)	11.6 (5.52)	11.4 (4.28)	11.5 (4.78)	11.3 (3.89)	0.08
FOR	0.52 (0.078)	0.50 (0.072)	0.51 (0.075)	0.53 (0.10)	0.54 (0.11)	0.53 (0.11)	0.52 (0.09)	0.03
CSF diversion	*n* (%)			*n* (%)				
Required	20 (26.0%)	25 (29.4%)	45 (27.8%)	34 (65.4%)	60 (73.2%)	94 (70.1%)	139 (47.0%)	** < 0.01**
VP shunt	8 (10.4%)	18 (21.2%)	26 (16.0%)	33 (63.5 % 0	58 (70.7%)	91 (67.9%)	117 (39.5%)	** < 0.01**
ETV	4 (5.2%)	4 (4.7%)	8 (4.9%)	0 (0%)	1 (1.2%)	1 (0.7%)	9 (3.0%)	
VP shunt and ETV	8 (10.4%)	3 (3.5%)	11 (6.8%)	1 (1.9%)	1 (1.2%)	2 (1.5%)	13 (4.4%)	
Age (mean avg.)	wks (SD)			wks (SD)				
Gestational age at delivery (wks)	35.4 (2.32)	35.4 (2.60)	35.4 (2.46)	38.0 (1.09)	37.0 (2.17)	37.4 (1.89)	36.0 (2.45)	** < 0.01**
Age at first CSF diversion (wks)	24.0 (14.2)	24.4 (23.8)	24.2 (19.9)	1.74 (1.88)	3.45 (8.19)	2.83 (6.67)	9.74 (16.0)	** < 0.01**

Abbreviations: *AD* atrial diameter, *MRI* magnetic resonance imaging, *SD* standard deviation, *US* ultrasound, *FOR* frontal occipital horn ratio, *VP* ventriculoperitoneal shunt (this category includes ventriculoatrial and ventriculopleural shunts as well), *ETV* endoscopic third ventriculostomy, *CSF* cerebrospinal fluid

Of the 151 postnatal MMC/MS patients, 13 were excluded due to defect type (lipomyelocystocele, meningocele, or myelocystocele), one was excluded for holoprosencephaly, and three were excluded for lack of prenatal evaluation. Of the remaining 134, eight lacked an FOR measurement and two had their first FOR measured after 7 days of age (Fig. 1).

At the time of delivery, fetal patients had a lower average gestational age (35.4 weeks) compared to their postnatal counterparts (37.4 weeks, *p* < 0.01; Table 1). The level of spinal defect for fMMC/MS patients was at T12 and above in 1.2%, L1 - 3 in 51.9%, and L4 or below in 46.9% (Table 1). For pMMC/MS patients, 12.7% had lesions at T12 and above, 32.8% at L1 - 3, and 54.5% at L4 and below (Table 1). There were no differences between groups. Talipes equinovarus of the feet was seen in 20.4% of fetal patients compared to 24.6% for postnatal patients (*p* = 0.46).

### Incidence of hydrocephalus and CSF diversion

AD, as measured on both prenatal MRI and prenatal ultrasound, was similar between the two cohorts (*p* = 0.27 and 0.08, respectively; Table 1). As measured on a postnatal ultrasound within the first week of life, FOR was smaller for fetal patients (0.51 ± 0.075) than postnatal patients (0.53 ± 0.11) after correcting for multiple comparisons (*p* = 0.03; Table 1).

Patients who underwent pMMC/MS closure were more likely to require CSF diversion (70.1%) than their fMMC/MS counterparts (27.8%, *p* < 0.01; Table 1). Within the fMMC/MS group, the rate of CSF diversion was also affected by the size of the ventricles at time of fetal repair. Patients with AD < 10 mm were treated for hydrocephalus 16.1% of the time, patients with AD 10–15 were treated 26.8% of the time, and patients with ventricles > 15 were treated 56.5% of the time (*p* < 0.01; Table 2).

**Table 2:** Ventriculometric correlation of CSF diversion dependence

	Prenatal AD (MRI)						*p* value
	< 10 mm (*n* = 56)		10–15 mm (*n* = 82)		> 15 mm (*n* = 23)		
	Mean (SD)	Median [min, max]	Mean (SD)	Median [min, max]	Mean (SD)	Median [min, max]	
AD	8.19 (1.14)	8.9 [5.0, 9.5]	12.8 (1.52)	13 [10.0, 15.0]	16.4 (1.72)	17.0 [15.5,22.0]	
CSF diversion	16.1% (*n* = 9)		26.8% (*n* = 22)		56.5% (*n* = 13)		< 0.01
Timing of CSF diversion (mo)	9.1 (7.4)	8.3 [1.6, 26.2]	4.8 (3.1)	4.6 [0.06, 12.5]	4.6 (3.2)	5.4 [0.1, 9.1]	0.01
	Postnatal FOR						
	< 0.5 (*n* = 63)			≥ 0.5 (*n* = 77)			
	Mean (SD)	Median [min, max]		Mean (SD)		Median [min, max]	
FOR	0.45 (0.03)	0.45 [0.33, 0.49]		0.56 (0.06)		0.54 [0.50,0.78]	
CSF diversion	14.3% (*n* = 9)			41.6% (*n* = 32)			< 0.01
Timing of CSF diversion (mo)	10.0 (7.2)	9.0 [1.6, 26.7]		4.2 (2.8)		3.8 [0.07, 10.1]	< 0.01

Abbreviations: *AD* atrial diameter, *MRI* magnetic resonance imaging, *SD* standard deviation, *CSF* cerebrospinal fluid, *FOR* frontal occipital horn ratio

Interestingly, patients who underwent fetal repair did not require CSF diversion until an average of age 24.2 ± 19.9 weeks, as opposed to 2.83 ± 6.6 weeks for pMMC/MS patients (*p* < 0.01; Table 1). The timing of shunt placement was also affected by the size of the ventricles on prenatal imaging for fMMC/MS patients. In fetal repaired patients with AD < 10 mm, treatment occurred at 9.1 months (SD 7.4), compared to 4.8 months (SD 3.1) in patients with AD 10–15 mm and 4.6 months (SD 3.2) in patients with AD > 15 (*p* = 0.01; Table 2). This demonstrates fetal AD impacts both the need and the timing for hydrocephalus treatment.

### Progression of hydrocephalus development in fMMC/MS patients

Best-fit head circumference growth curves were generated for fMMC/MS repair patients. This included 4024 unique measurements across the 162 fMMC/MS patients. The cohort was further stratified by patient sex (77 female, 1856 measurements; 85 male, 2168 measurements) and those who required VP shunt or ETV versus those who remained CSF diversion independent. These groups were examined separately to discern differences in patterns of hydrocephalus development among this unique clinical group.

**Fig. 2:**
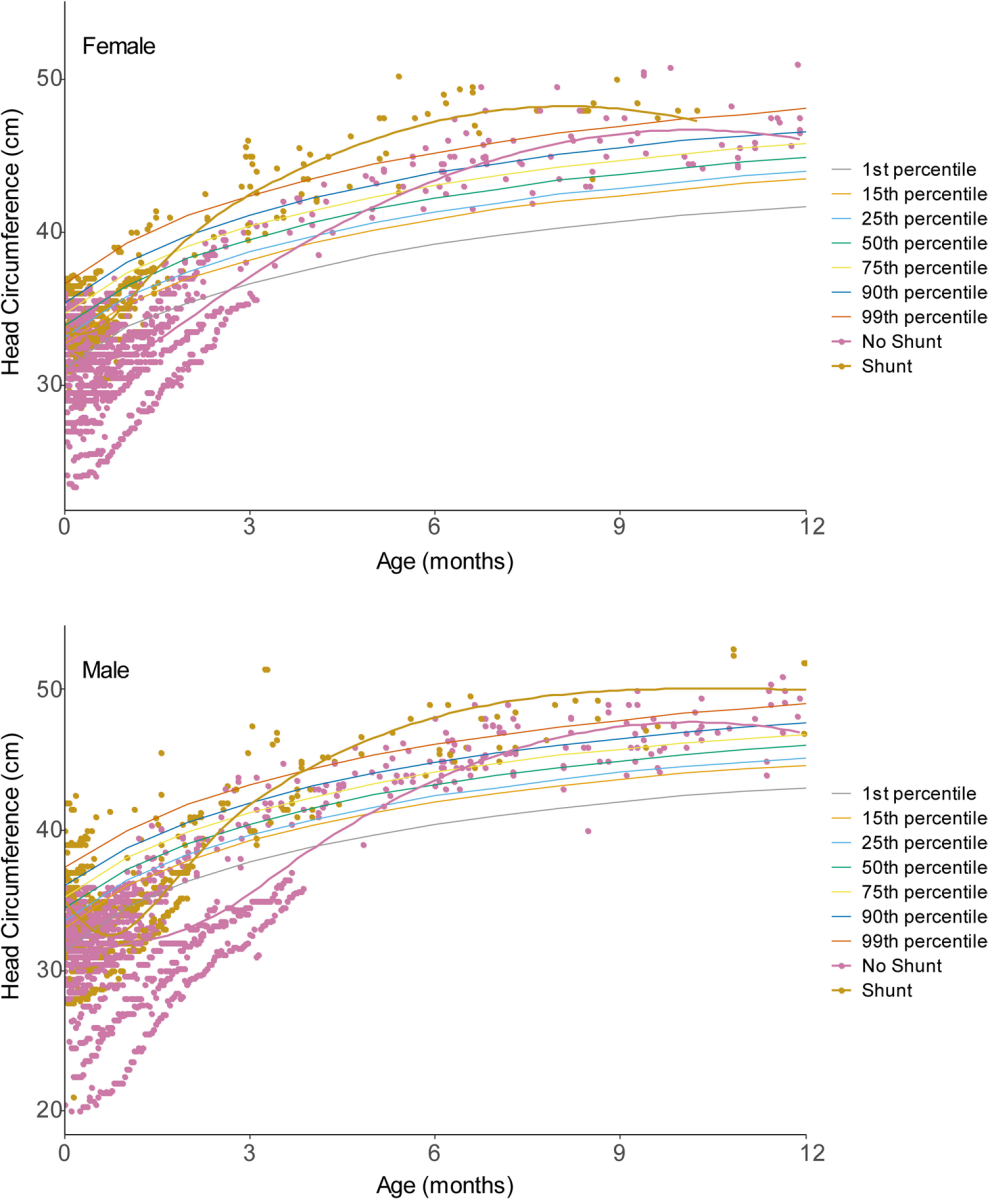
Shunted and non-shunted patients’ head circumference as a function of age. Female (top) and male (bottom) patients’ head circumference (HC) are plotted as a function of time. The orange curve is a best-fit representation of those patients who required CSF diversion, whereas the pink curve is a best-fit representation of those patients who did not require CSF diversion. Normalized HC percentiles are represented behind the data. The female graph is composed of 1856 measurements from 77 unique patients. The male graph is composed of 85 male, 2168 measurements

In both males and females, patients who required CSF diversion crossed the 99 th percentile for HC. This occurred around 4 months of age for males and 3.5 months of age for females (Fig. 2). These timepoints unsurprisingly approach the average age of shunt placement (5 months) in the fMMC/MS group. Those who never required CSF diversion on average never crossed the 99 th percentile for HC, and their curve is right shifted as compared to the group requiring CSF diversion (Fig. 2).

### Imaging as a predictor of hydrocephalus development in fMMC/MS patients

**Fig. 3:**
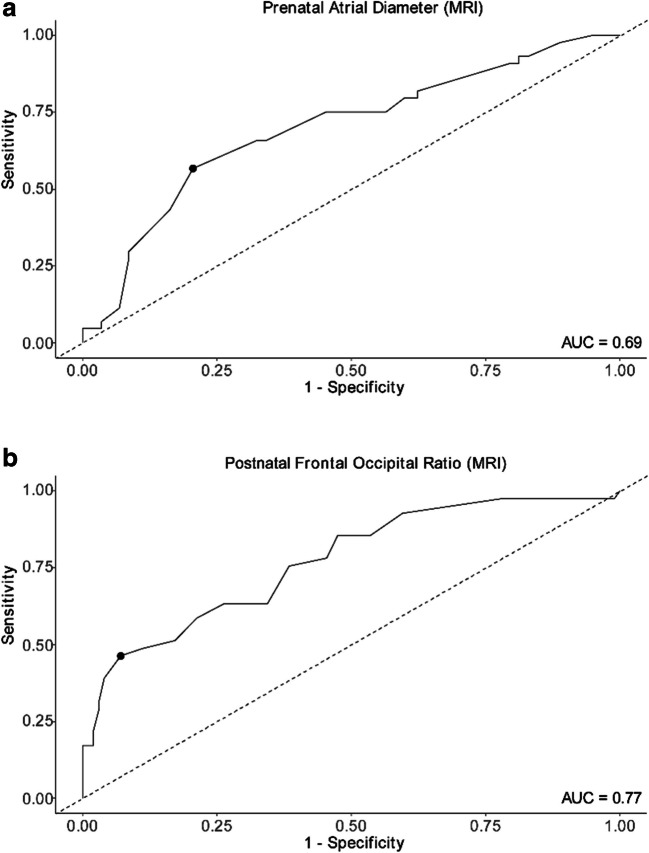
Receiver operating characteristic (ROC) curves of **a** prenatal atrial diameter and **b** postnatal frontal occipital ratio for predicting need for cerebrospinal fluid diversion. Receiver operating characteristic (ROC) curves illustrating the predictive accuracy of **a** prenatal atrial diameter (AD) and **b** postnatal frontal occipital ratio (FOR), both measured via MRI. The optimal cut points for each metric, determined by Youden’s index analysis, are indicated with dots on the respective ROC curves. The area under the curve (AUC) indicates the discriminatory power of each measurement

fMMC/MS patients with FOR ≥ 0.5 had higher rates of CSF diversion than those < 0.5 (41.6%, *n* = 32 vs 14.3%, *n* = 9; *p* < 0.01) (Table 2). Given that both prenatal AD and postnatal FOR correlated with the need for CSF diversion, Youden’s index was calculated to assess this index strength as a predictor of shunt placement. In brief, Youden’s cut point analysis maximizes the difference between the true positive and false positive rates to create a meaningful cutoff within a cohort (refer to methods). Youden’s analysis yielded an optimal cut point to predict shunt dependence of 14 mm for prenatal MRI AD (sensitivity 0.57, specificity 0.79, Youden’s index 0.36; Fig. 3a). When evaluating FOR calculated from postnatal imaging in the first week of life with the same outcome, Youden’s analysis yielded a cut point of 0.57 (sensitivity 0.46, specificity 0.93, Youden index 0.39; Fig. 3b).

## Discussion

The findings from this study provide valuable insights into the development of hydrocephalus and predictors of CSF diversion dependence in patients undergoing fMMC/MS repair in the post-MOMS era. Consistent with the results of the MOMS trial, we redemonstrate that patients undergoing fMMC/MS repair develop hydrocephalus at a later age and have lower rates of shunt placement compared to the pMMC/MS counterparts. However, our data demonstrate an even lower rate of shunting for the fMMC/MS cohort as compared to the MOMS trial [16]. Extending this knowledge further, we show that some early ventriculometrics help to predict both the need and the timing of hydrocephalus treatment. fMMC/MS patients who will eventually progress to require shunt or ETV also follow a distinct head circumference growth curve. In totality, these data suggest that patients who undergo fMMC/MS repair are clinically distinct from other neonates from the perspective of hydrocephalus development.

### Comparative outcomes of fMMC/MS repair versus pMMC/MS closure

Our data demonstrate that patients who undergo fMMC/MS repair require significantly less CSF diversion compared to those in the pMMC/MS cohort (27.8% vs. 70.1%, *p* < 0.01). This difference supports, and actually exceeds, the benefit demonstrated in previous studies [12, 16–18]. One factor contributing to this reduction in shunt dependence may be the evolution of our institutional closure technique. We now employ myofascial flaps that was not done in the original MOMS study [19]. The protective effect that fMMC/MS has on hydrocephalus development is attributed to two mechanisms: (1) decreased CSF leak through the fetal defect restores spinal canal CSF pressure, improves hydrodynamics, and results in ascent of the developing hindbrain thereby reducing traction on CSF outflow tracts; (2) reduced duration of in utero exposure to amniotic fluid, which is known to have a toxic effect on the developing nervous system [2–4]. Myofascial flaps may provide a more robust barrier between amniotic fluid and neural structure as the fetus heals after intrauterine repair.

Patients in the pMMC/MS cohort underwent shunt placement at a younger age (2.83 ± 6.67 weeks) than their fMMC/MS counterparts (24.2 ± 19.9 weeks). To further explore hydrocephalus development in the fetal cohort given their unique clinical features, we generated a best-fit head circumference growth curve. The fMMC/MS repair group has a delayed upslope of HC growth around 4–5 months of age, compared to the pMMC/MS group who developed hydrocephalus just days to weeks postoperatively. These data concur with Karuparti et al. who also investigated CSF diversion timing and modeled HC in fMMC/MS and pMMC/MS patients. They found that fMMC/MS patients presented with delayed hydrocephalus—albeit not as delayed as our data—and that a subgroup of pMMC/MS patients developed hydrocephalus with an early, rapid increase in HC [20, 21]. It is possible that the severity of hydrocephalus is simply greater in the postnatal cohort leading to its early clinical presentation. Particularly in cases with more severe hindbrain herniation, obstructive physiology may lead to a more rapid increase in HC. Graphics in Fig. 2 should serve as a prognostic tool for spina bifida care givers with patients who are tracking towards clinical hydrocephalus.

This delayed hydrocephalus presentation in fetal repaired patients has potential benefits. The older age at time of shunt placement may reduce complication rates, ultimately decreasing the number of revisions in the neonatal period [22]. Additionally, patients that present with hydrocephlus later in life have higher success rates for endoscopic third ventriculostomy (ETV) [23]. Further study into CSF diversion complications as a function of closure method will shed light on this hypothesis. A potential challenge of delayed hydrocephalus presentation may be its timely identification. Given that most fetal repair patients will be discharged from the hospital by the time hydrocephalus develops, an increased vigilance is required by pediatricians and neurosurgeons alike.

### Early ventriculo/cranio-metrics as a predictor of shunt dependence

For the purposes of enhancing prenatal counseling and prognostication, we explored fetal neuroimaging characteristics. Within our fMMC/MS cohort, patients with a high prenatal AD (> 10 mm) or FOR (≥ 0.5) had higher rates of CSF diversion (*p* < 0.01) than those with lower values. Alternative cut points for discriminating between “high” and “low” AD and FOR values were derived using Youden’s index method and were similarly able to predict eventual shunt dependence with fair sensitivity (0.57 and 0.46) and high specificity (0.79 and 0.93).

These findings are unsurprising given that ventriculomegaly is one criterion to consider when evaluating for shunt placement, and thus, it may reflect the clinician’s behavior as much as it represents a predictive variable. However, despite these predictable trends, these data alone do not help us to define objective metrics by which to strongly consider shunt placement. Youden’s index goes one step beyond simply defining trends and suggests an optimal cut point for predicting CSF diversion dependence from early ventriculometrics, with a prenatal AD cut point of 14 mm and a postnatal FOR cut point of 0.57. These cut points could serve as practical clinical tools for early identification of patients at greater risk for hydrocephalus.

### Clinical implications

The findings from this study support the continued utilization of fMMC/MS repair as a standard of care for those who meet eligibility criteria, given its association with reduced rates of CSF diversion and delayed time to shunting. Moreover, our identification of specific early imaging markers (AD and FOR) that predict the need for CSF diversion could enhance prenatal counseling and postnatal prognostication. While these measurements may stratify patients into high- and low-risk categories for the development of hydrocephalus, our data emphasize the importance of long-term follow-up for all fMMC/MS repair patients, as the timeline of their hydrocephalus progression is delayed compared to those who undergo pMMC/MS closure. Frequent head circumference measurements and cranial imaging should be standard of care for this population, even in the absence of any immediate postnatal concerns. Recent studies have identified further parameters, such as clivus-supraocciput angle and transcerebellar diameter as a measure for hindbrain herniation, that are not routinely measured during routine clinical care [24, 25]. These studies are limited by small sample size; however, they provide valuable insight into variables that may be incorporated into future prognostication models. Future research should aim to refine these predictive markers and explore additional factors that may influence long-term outcomes in MMC patients. Additionally, ongoing evaluation of the neurodevelopmental and functional outcomes of patients who undergo fetal MMC/MS repair is essential to fully understand the benefits and potential risks of this intervention.

## Limitations

This study has several limitations that should be considered. The retrospective nature of the analysis and the reliance on a single institution's data may limit the generalizability of the findings. Parental preferences and exclusion criteria for prenatal repair versus postnatal closure may have also biased the data. Conducting these studies withing larger, collaborative groups such as the Hydrocephalus Clinical Research Network, North American Fetal Therapy Network, and the Children’s Hospital Neonatal Consortium are critical for creating the most applicable prognostic models possible.

## Conclusion

Our study reaffirms the advantages of fetal MMC/MS closure in reducing the need for CSF diversion and provides valuable predictors for early identification of patients at risk for hydrocephalus. By integrating these findings into clinical practice, healthcare providers can improve the management and outcomes of patients with MMC/MS, ultimately enhancing their quality of life. Further multicenter studies and long-term follow-up are needed to validate these results and optimize care strategies for this vulnerable population.

## Data Availability

Data used within this manuscript is housed within the Clinical Outcomes Data Archive at the Children's Hospital of Philadelphia and is available upon request of the corresponding and senior authors (kundishora@chop.edu; heuerg@chop.edu).

## References

[1] Marcati E, Meccariello G, Mastino L, Picano M, Giorgi PD, Talamonti G (2024) Myelomeningocele: long-term neurosurgical management. Adv Tech Stand Neurosurg. 10.1007/978-3-031-42398-7_6 10.1007/978-3-031-42398-7_6 38700682

[2] Meuli M, Meuli-Simmen C, Hutchins GM, Yingling CD, Hoffman KM, Harrison MR, Adzick NS, Meuli M, Meuli-Simmen C, Hutchins GM, Yingling CD, Hoffman KM, Harrison MR, Adzick NS (1995) In utero surgery rescues neurological function at birth in sheep with spina bifida. Nat Med 1(4):1 7585064 10.1038/nm0495-342

[3] Drewek MJ, Bruner JP, Whetsell WO, Tulipan N (1997) Quantitative analysis of the toxicity of human amniotic fluid to cultured rat spinal cord. Pediatr Neurosurg 27. 10.1159/000121250 10.1159/000121250 9577972

[4] Athiel Y, Jouannic J-M, Lépine M, Maillet C, Denis TdS, Larghero J, Guilbaud L (2024) Role of amniotic fluid toxicity in the pathophysiology of myelomeningocele: a narrative literature review. Prenat Diagn. 10.1002/pd.6681 10.1002/pd.6681 39370541

[5] Parker SE, Mai CT, Canfield MA, Rickard R, Wang Y, Meyer RE, Anderson P, Mason CA, Collins JS, Kirby RS, Correa A (2010) Updated national birth prevalence estimates for selected birth defects in the United States, 2004–2006. Birth Defects Res A Clin Mol Teratol 88. 10.1002/bdra.20735 10.1002/bdra.20735 20878909

[6] Burke R, Liptak GS, Disabilities tCoCW (2011) Providing a primary care medical home for children and youth with spina bifida. Pediatrics 128. 10.1542/peds.2011-2219 10.1542/peds.2011-2219 22123894

[7] Shin M, Kucik JE, Siffel C, Lu C, Shaw GM, Canfield MA, Correa A (2012) Improved survival among children with spina bifida in the United States. J Pediatr 161 10.1016/j.jpeds.2012.05.040 10.1016/j.jpeds.2012.05.040 PMC4547557 22727874

[8] Meller C, Covini D, Aiello H, Izbizky G, Medina SP, Otaño L (2021) Update on prenatal diagnosis and fetal surgery for myelomeningocele. Arch Argent Pediatr 119. 10.5546/aap.2021.eng.e215 10.5546/aap.2021.eng.e215 34033426

[9] Yamashiro KJ, Farmer DL (2021) Fetal myelomeningocele repair: a narrative review of the history, current controversies and future directions. Transl Pediatr 10. 10.21037/tp-20-87 10.21037/tp-20-87 PMC8192992 34189108

[10] Sutton LN, Adzick NS, Bilaniuk LT, Johnson MP, Crombleholme TM, Flake AW (1999) Improvement in hindbrain herniation demonstrated by serial fetal magnetic resonance imaging following fetal surgery for myelomeningocele. JAMA 282:1826–1831 10573273 10.1001/jama.282.19.1826

[11] Bouchard S, Davey MG, Rintoul NE, Walsh DS, Rorke LB, Adzick NS (2003) Correction of hindbrain herniation and anatomy of the vermis after in utero repair of myelomeningocele in sheep. J Pediatr Surg 38. 10.1053/jpsu.2003.50078 10.1053/jpsu.2003.50078 12632366

[12] Adzick NS, Thom EA, Spong CY, John W. Brock I, Burrows PK, Johnson MP, Howell LJ, Farrell JA, Dabrowiak ME, Sutton LN, Gupta N, Tulipan NB, D'Alton ME, Farmer DL (2011) A randomized trial of prenatal versus postnatal repair of myelomeningocele. N Engl J Med 364. 10.1056/NEJMoa1014379 10.1056/NEJMoa1014379 PMC3770179 21306277

[13] Rocque BG, Bishop ER, Scogin MA, Hopson BD, Arynchyna AA, Boddiford CJ, Shannon CN, Blount JP (2015) Assessing health-related quality of life in children with spina bifida. J Neurosurg Pediatr 15:144–149 25415252 10.3171/2014.10.PEDS1441

[14] Karmur BS, Kulkarni AV (2018) Medical and socioeconomic predictors of quality of life in myelomeningocele patients with shunted hydrocephalus. Childs Nerv Syst 34:741–747 29249073 10.1007/s00381-017-3691-8

[15] Reynolds TA, Goldshore MA, Flohr S, Land S, Mathew L, Gebb JS, Oliver ER, Rintoul NE, Ades AM, Foglia EE, Avitabile CM, Panitch HB, Heuer GG, Howell LJ, Adzick NS, Hedrick HL (2024) A clinical outcomes data archive for a comprehensive fetal diagnosis and treatment center. Fetal Diagn Ther: 1–9. 10.1159/000541877 10.1159/000541877 39378854

[16] Tulipan N, Wellons JC 3rd, Thom EA, Gupta N, Sutton LN, Burrows PK, Farmer D, Walsh W, Johnson MP, Rand L, Tolivaisa S, D’Alton ME, Adzick NS, Investigators M (2015) Prenatal surgery for myelomeningocele and the need for cerebrospinal fluid shunt placement. J Neurosurg Pediatr 16:613–620 26369371 10.3171/2015.7.PEDS15336 PMC5206797

[17] Flanders TM, Heuer GG, Madsen PJ, Buch VP, Mackell CM, Alexander EE, Moldenhauer JS, Zarnow DM, Flake AW, Adzick NS (2020) Detailed analysis of hydrocephalus and hindbrain herniation after prenatal and postnatal myelomeningocele closure: report from a single institution. Neurosurgery 86:637–645 31432079 10.1093/neuros/nyz302

[18] Farmer DL, Thom EA, Brock JW 3rd, Burrows PK, Johnson MP, Howell LJ, Farrell JA, Gupta N, Adzick NS, Management of Myelomeningocele Study I (2018) The management of myelomeningocele study: full cohort 30-month pediatric outcomes. Am J Obstet Gynecol 218(256):e251-256 e213 10.1016/j.ajog.2017.12.001 PMC7737375 29246577

[19] Flanders TM, Madsen PJ, Pisapia JM, Hudgins ED, Mackell CM, Alexander EE, Moldenhauer JS, Zarnow DM, Flake AW, Adzick NS, Heuer GG (2020) Improved postoperative metrics with modified myofascial closure in fetal myelomeningocele repair. Oper Neurosurg (Hagerstown) 18:158–165 10.1093/ons/opz115 31222267

[20] Karuparti S, Flanders TM, Dunbar A, Varagur K, Strahle JM (2024) Head growth in patients with myelomeningocele treated with prenatal and postnatal surgery. J Neurosurg Pediatr 33:554–563 38457805 10.3171/2023.11.PEDS23328

[21] Karuparti S, Dunbar A, Varagur K, Sudanagunta K, Mingo M, Bligard KH, Odibo A, Vrecenak J, McEvoy S, Limbrick D, Peglar Marsala L, Anadkat J, Mian A, Strahle JM (2024) Predictors and timing of hydrocephalus treatment in patients undergoing prenatal versus postnatal surgery for myelomeningocele. J Neurosurg Pediatr 33:544–553 38457812 10.3171/2023.10.PEDS23327

[22] Dupepe EB, Hopson B, Johnston JM, Rozzelle CJ, Jerry Oakes W, Blount JP, Rocque BG (2016) Rate of shunt revision as a function of age in patients with shunted hydrocephalus due to myelomeningocele. Neurosurg Focus 41:E6 27798984 10.3171/2016.8.FOCUS16257 PMC5460762

[23] Kulkarni AV, Drake JM, Mallucci CL, Sgouros S, Roth J, Constantini S, Canadian Pediatric Neurosurgery Study G (2009) Endoscopic third ventriculostomy in the treatment of childhood hydrocephalus. J Pediatr 155:254-259 e251 19446842 10.1016/j.jpeds.2009.02.048

[24] Kunpalin Y, Sichitiu J, Krishnan P, Blaser S, Kulkarni AV, Abbasi N, Ryan G, Shinar S, van Mieghem T (2023) Simple prenatal imaging predictors for postnatal cerebrospinal fluid diversion surgery in fetuses undergoing in utero surgery for spina bifida. Prenat Diagn 43:1605–1613 37975651 10.1002/pd.6453

[25] Mangano FT, Altaye M, Stevenson CB, Yuan W (2022) The construction of a predictive composite index for decision-making of CSF diversion surgery in pediatric patients following prenatal myelomeningocele repair. AJNR Am J Neuroradiol 43:1214–1221 35902125 10.3174/ajnr.A7585 PMC9575433

